# Pragmatics Always Matters: An Expanded Vision of Experimental Pragmatics

**DOI:** 10.3389/fpsyg.2020.01619

**Published:** 2020-07-24

**Authors:** Raymond W. Gibbs, Herbert L. Colston

**Affiliations:** ^1^Independent Researcher, Soquel, CA, United States; ^2^Department of Linguistics, University of Alberta, Edmonton, AB, Canada

**Keywords:** pragmatics, experimental pragmatics, individual differences, task demands, psycholinguistics

## Abstract

Much of the work in experimental pragmatics is devoted to testing empirical hypotheses that arise within the study of linguistic and philosophical pragmatics. The focus in much of this work is focused on those aspects of communicated meaning that are “inferred” rather than understood through linguistic “coding” processes. Under this view, pragmatic meanings emerge secondarily after purely linguistic meanings are accessed or computed. Our aim in this article is to greatly broaden the scope of experimental pragmatic studies by calling for much greater emphasis on the complete pragmatics of language use. Pragmatics is continuously present and constrains people’s real-time production and processing of language in context. Experimental pragmatics should attend more to the particularities of pragmatic experience through closer examination of the people we study, the specific tasks used to assess understanding, as well as the actual complex meanings people interpret in diverse contexts. The many specifics of human pragmatics demand the study and theoretical inclusion of many bodily, linguistic, and situational factors that make up each instance of meaning making.

## Introduction

Experimental pragmatics has had a complex history in its 40 or so years of existence. The field emerged back in the 1970s as various psychologists, both those studying developmental psychology and psycholinguistics, as well as linguistics, began to explore people’s understandings of pragmatic meaning, which was quite a departure from the traditional emphasis in psycholinguistics on lexical, syntactic, and semantic processing of individual sentence meaning. Certain critics within linguistics and psychology were skeptical about the possibility of scientifically examining pragmatic language production and interpretation. One often repeated refrain from the 1970s and 1980s was that “pragmatics is the wastebasket of linguistics,” a claim that suggests the impossibility of making proper scientific order out of a human endeavor which is so messy and intractable. Still, psycholinguists found much inspiration, and even testable hypotheses, in the writings of linguists and philosophers interested in pragmatics (Clark, 1996; Noveck and Sperber, 2004; Bara, 2010; Noveck, 2018; Gibbs, 2019). The field of experimental pragmatics has continued to survive, and make its mark, within the larger interdisciplinary world of cognitive science.

Many practitioners of experimental pragmatics see their work as explicitly devoted to testing the claims of those studying linguistic and philosophical pragmatics. A tremendous body of experimental work has spoken positively and negatively about different facets of various linguistic pragmatic theories (Noveck, 2018; Huang, 2019). One lingering assumption in much experimental pragmatics research is the idea that “pragmatics” refers, somewhat narrowly, to those aspects of linguistic processing that are inferential, and not due to temporarily earlier linguistic coding/decoding processes. Under this view, people begin understanding what speakers mean by first engaging in many fast-acting linguistic processes in which sounds are recognized and then syntactic and semantic analyses are completed. Pragmatic meaning is created later on via special pragmatic inferential processes that may be generally applied to all utterances or are optionally applied given specific forms of linguistic input (e.g., different processes are needed to determine metaphor as opposed to ironic speaker meaning) (e.g., the standard pragmatic model, see Gibbs, 1994). Noveck (2018) argues that part of this view is motivated by ideas about modularity within cognitive science, more generally.

A related emphasis in experimental pragmatics is on the role that “theory of mind” or “mind-reading” plays in pragmatic language interpretation (Noveck, 2018). The focus here has been to explore the ways that understanding what people say or write depends on creating a theory of that person’s mind, or specific thoughts in some communicative situation (Nichols and Stich, 2003). Experimental studies on theory of mind in pragmatic interpretation have examined a number of ways that people’s cognitive abilities, and sometimes inabilities, to infer speakers’ possible mental states are a critical facet of interpersonal communication (Kissine, 2016; Bosco et al., 2018). Some pragmatic theories go so far as to suggest that there is a “relevance theoretic comprehension procedure” module that is embedded within a larger “theory of mind” module (Sperber and Wilson, 2002).

Our argument in this article is that these traditional views on pragmatic meaning, despite their contributions to experimental pragmatics, under-estimate the true, and complex reality of pragmatic meaning making. We maintain that experimental pragmatics should be more than the testing of ideas from linguistic pragmatic theory. Experimental investigations must pay much greater attention to the larger ways that pragmatics always shapes our use and understanding of both linguistic and non-linguistic meanings, as seen in research on multimodal communication (Shockley et al., 2009; Hollers and Levinson, 2019). Pragmatics is much greater than the study of particular inferential processing stages, because people are always doing pragmatics within each moment of their lives. This includes people’s pragmatic participation in experimental studies. We suggest the need for an expanded vision of experimental pragmatics, one that extends more deeply into the different ways that our doing pragmatics shape experimental participants’ performances. Pragmatics is not merely a specific type of inferential processing, and it is not just a type of knowledge that differs from that accessed during various parts of language production and processing (e.g., lexicon, grammar, and semantics). Pragmatics is more fundamentally the entirety of people’s adaptive performances in varying circumstances and contexts.

This article discusses several research practices within the field of experimental pragmatics over the last few decades. Our aim is not to criticize particular people. Both of us have engaged in some of the practices we take issue with in what follows. Some readers may also suggest that the situation we outline is not as bad as we make it out to be. Our aim, though, is to encourage discussion and debate in order to move experimental pragmatics studies forward to more adequately addressing “pragmatics” in a broader, psychologically real, fashion than it has been in the past.

## The Problem

Experimental pragmatics studies typically explore what kinds of pragmatic processing emerges at what points during people’s use and interpretation of language. Early theories in the field often assumed that pragmatic knowledge and inferential processes were recruited relatively late in the understanding process, especially when compared to the access of other sources of linguistic information (e.g., lexical, syntactic, and semantic) (see Gibbs, 1994; Gibbs and Colston, 2012). But the strong trend in experimental findings over the last several decades shows that pragmatic knowledge and pragmatic inferences comes into play very early during the online interpretation of language in context (Gibbs, 1994, 2019; Noveck and Sperber, 2004). People do not perform purely linguistic analyses first on a word string and only later recruit pragmatics to infer what speakers/writers aim to communicate. Instead, pragmatics has its influence through the immediate, automatic construction of what people imply by the words they speak and write (Gibbs, 1994; Gibbs and Colston, 2012). Pragmatics does not come into play only at certain temporal points in language use, and is not turned on and off in people’s linguistic and non-linguistic experiences. Theoretical models in psycholinguistics now mostly embrace the idea that pragmatics, often through access to prior pragmatic background knowledge and more proximate contextual information, constrains all facets of the understanding process (Campbell and Katz, 2012; McRae and Matsuki, 2013; McClelland et al., 2014).

Our concern, however, is with two unacknowledged assumptions in the traditional study of experimental pragmatics. First, there is surprisingly little discussion of what it really means to say that some pragmatic message (e.g., “This soup needs salt” implies “Pass me the salt”) has been “understood.” Pragmatic understanding is assumed to be a general goal that all people in all contexts aim to achieve. But people differ in their cognitive and personal make-up, as well as their understanding motivations, in various circumstances. These individual variations, both between and within people, are critical to take into account in any theoretical characterization of how people interpret pragmatic messages.

Second, experimental pragmatics examines people’s language understanding abilities by asking participants to perform a wide range of experimental tasks. These task demands constitute a big part of the inherent pragmatics within any experimental study (e.g., developmental studies have long struggled with how implicit and explicit task demands affect behavioral outcomes in cognitive and linguistic studies). Yet this aspect of pragmatic experience is not sufficiently acknowledged in scholars’ theoretical interpretations of experimental results within psycholinguistics and cognitive neuroscience. As is often the case in experimental studies of human perception and cognition, we too often strip away the task demands in creating theories of pragmatics as if this critical feature of experimental studies is irrelevant to characterizing the role that pragmatics has in people’s use and understanding of language in context.

In addition to these difficulties, there is also the problem that experimental pragmatics focuses mostly on the “processes” by which language is acquired, produced, and understood, but is far less dedicated to explaining meaning “products” that people really convey or interpret in real-world language situations. The relative neglect of pragmatic “products” in experimental pragmatics comes with a great cost. We too often assume that people experience a definitive “click of comprehension” when pragmatic messages are singularly encountered and understood. Yet this mistakenly assumes that experimental pragmatics should focus on the use and understanding of different types of pragmatic meanings (e.g., scalar implicatures, presuppositions, politeness, negation, and metaphor), but not the very specific tokens of meaning that people may often infer in discourse. This difficulty also alerts us to the need to significantly broaden our vision of pragmatics by looking more closely at what participants are fully engaged in during different experimental situations.

## Individual Differences

Most theories within linguistic pragmatics offer detailed proposals on the ways ordinary people use and understand pragmatic meanings (Huang, 2019). These theoretical proposals typically assume some idealized speaker/hearer who is an adult possessing relatively intact neural, cognitive and linguistic abilities. Of course, there is an extensive body of research looking at variations in pragmatic language talents, such as children who are still acquiring pragmatic language skills, and atypical children and adults who may be limited because of brain injury, disease (e.g., Alzheimer’s) or developmental disorders (e.g., autism) (Cummings, 2019). The classic assumption, nonetheless, is that differences in pragmatic language performances are mostly evidence of pragmatic deficits in which the typical, normative module of pragmatic competence is not functioning as expected.

But there exists a range of evidence showing important individual differences that shape pragmatic performances in experimental pragmatic studies. For example, there is an emerging body of research showing many variations within, and between, experimental participants. Consider some of the individual differences that have been empirically shown to influence figurative language use and understanding, including language experience, gender, occupation, social status and culture, political background/beliefs, cognitive differences (e.g., IQ, working memory capacity), bodily action, geographic origin, personality, social relationship, and common ground (Gibbs and Colston, 2012). These factors have their assorted influences on both the processing of figurative language, such as metaphor and irony, and the exact meaning products people infer when they encounter different tropes in various experimental situations.

Many scholars in experimental pragmatics may argue that it should be possible to control for, or factor away, individual variations in order to create normative theories of pragmatic language abilities without regard to complex arrays of individual differences. Our reply is that trying to control for, and then eliminate the need to account for, individual differences turns a blind eye to the real complexities of pragmatic experiences. Individual differences are not mere representations of “noise” around some normative mechanism of pragmatic meaning understanding. The fact of the matter is that individual differences always have a critical role in the psychology of pragmatic behaviors.

There are also within-individual variations that affect pragmatic performances in experimental situations. For example, a typical study in experimental pragmatics will present individual participants a set of stimuli, representing different independent variables, which they will respond to in some instructed manner. We often compute averages of people’s behavioral performances across the many stimuli in each experimental condition. The aim here is to capture something about the central tendencies in people’s reactions to different experimental conditions and looking at means is widely viewed as the most appropriate descriptive statistic by which to achieve this goal.

But means or averages hide the fuller complexity of people’s pragmatic behaviors in experimental studies. There is a good deal of work within experimental psychology that demonstrates how individual people’s in-experimental performances vary in systematic ways (Raczaszek-Leonardi and Kelso, 2007; Gibbs and Van Orden, 2010). Looking at the distributions of responses, such as reading times, can offer more insightful explanations for people’s experimental performances, including the idea that people are behaving as self-organizing dynamical systems within the experiment (Gibbs and Van Orden, 2010; Gibbs, 2017). For this reason, we must be careful not to assume, as is too often done, that the independent variable must only be caused by a specific, isolated mechanism in mind (e.g., pragmatic competence). Many independent variables may only have partial, probabilistic influence on people’s behaviors in experimental pragmatic tasks (Gibbs and Santa Cruz, 2012).

Our point is that the data obtained in experimental pragmatic studies do not simply reflect people’s responses to different experimental conditions and the independent variables these are meant to tap into. Instead, people’s individual pragmatic behaviors in any experimental situation are subtly shaped by their specific bodies, cultural expectations, personalities, and histories (Paxton and Dale, 2017; Abney et al., 2018). Pragmatics is, in this way, always a part of experiments we conduct and the data obtained from these investigations.

## Experimental Tasks

It is challenging to characterize the diversity of tasks employed in experimental pragmatics (Jucker et al., 2018). Nonetheless, a typical study in experimental pragmatics will present participants with a set of stimuli to which they are to respond in one of many possible ways. Among the most widely used experimental techniques are full-sentence reading times, word-by-word reading times (including both moving-window and eye-movement measures), self-paced listening, paraphrase judgment response times, priming methods, mouse-tracking, eye-tracking in visual world environments, free recall, cued recall, mental imagery studies, summarization and paraphrase of meaning tasks, question answering, cooperative conversation tasks, bodily enactment tasks, and various brain scanning measures such as evoked-related potentials (ERPs) and functional magnetic resonance imaging (fMRI) methods.

Each of these experimental techniques presumably taps into how people “understand” pragmatic meaning. But these measures reflect different facets of pragmatic understanding. For example, full phrase or sentence reading time studies offer evidence on the total cognitive effort required to interpret a particular kind of pragmatic meaning at the phrasal or sentence level, such as a figurative utterance (e.g., metaphor, idiom, and irony) or other kinds of conversational implicature (e.g., scalar implicature). Methods examining the time it takes people to read individual words in linguistic expressions conveying different kinds of pragmatic meaning, via moving-window or eye-movement techniques, are useful for exploring local processing of specific word meanings in context. These online techniques, along with brain scanning measures such as ERPs, provide insights into the interaction of linguistic, social/pragmatic and cognitive knowledge during real-time pragmatic language understanding. Asking people to paraphrase the meanings of different pragmatic messages, rapidly judge suggested paraphrases of utterance meaning, or engage in specific task-related conversations provide evidence that enables scholars to characterize the meaning products understood when people process pragmatic meanings. Similarly, imagery tasks provide another method for exploring the contents of what people have understood having just quickly read or heard a specific kind of pragmatic message. Bodily engagement tasks, where people are asked to perform specific gestures or adopt different postures, are critical for investigating the role of embodied experience and action in creating pragmatic understandings of words, phrases, and longer stretches of discourse.

In general, no single method is capable of examining all facets of pragmatic understanding. Each technique may reveal different aspects of what happens during people’s inferring of pragmatic meanings. In some cases, these insights into pragmatic language processing are specific to particular temporal dimensions of the online construction of pragmatic meaning. For instance, word-based processing measures aim to assess more local pragmatic processing as experimental participants read or listen to linguistic messages word-by-word. Full-time reading and priming tasks are better able to assess more global aspects of pragmatic meaning understanding, such as when an overall message is understood (e.g., does this phrase, sentence, in context convey metaphorical meaning or a specific scalar implicature?).

Our concern here is that there is still an overwhelming tendency in the literature for scholars to make generalizations from their task-specific studies to larger, comprehensive theories of pragmatics. A vast number of studies on figurative language use employs an extensive range of experimental methods in which participants are instructed to engage in different tasks, such as fast, word-by-word reading, full phrase or sentence reading, making quick judgments on whether a particular figurative utterance makes sense, or fits into the previously read story context, or determine if an utterance conveys literal or some kind of figurative meaning (e.g., metaphorical and ironic), and whether a figurative utterance is apt or creative (Gibbs and Colston, 2012; Colston, 2015). Each of these dependent measures may affect participants’ “understanding” performances in experimental situations given the different forms of attention they must pay to the stimulus materials. The results of these varying studies, and the theoretical interpretations scholars offer for explaining these findings, will differ depending on the explicit task required of the participants in a study. Yet these task influences are rarely acknowledged in linguistic pragmatic theories.

One possible response to this concern is to place most credibility in those experimental findings that converge across different experimental tasks (i.e., converging operations) (Gibbs, 2019). But it may still be difficult, if not impossible, to find experimental results that are truly universal across various people, languages, cultures, and task demands (Kecskes, 2014). A related response would be to argue that those that have the *greatest* convergence across people and tasks should be given the most weight in theoretical debates. However, arguments based on the “weight” of empirical evidence may be far less satisfactory to scientists who demand reliability and consistency in experimental findings.

Another response to the task demand problem in experimental pragmatics is when individual scholars argue for the superiority of some task environments (e.g., measures of eye-movements) over others (e.g., full phrasal or sentence reading times). The arguments along this line typically suggest that some specific task measures are better indicators of “real-world” pragmatic language use than others. Experiments that employ those privileged methods should, under this view, be afforded the most weight in debates over the content of pragmatic theories. It is fair to observe, however, that this type of response to the task demand issue typically ends in complete empirical stalemates as different scholars merely embrace results from preferred methods while ignoring or dismissing findings obtained from less preferred experimental paradigms.

The alternative position that is part of our broader vision of experimental pragmatics suggests that pragmatic language use is always task-specific both in and outside of experimental studies. Pragmatic language processing is not a uniform activity that operates in a task-free manner. Speakers and listeners always approach any language interaction or situation with explicit or implicit goals in mind. For instance, a listener can hear a political speech and wonder, even if implicitly, as to whether or not the message conveyed was persuasive, or whether or not he/she appreciated what a speaker has stated or an author wrote. People listen to language hoping to remember what was stated, in some circumstances, and may, therefore, pay close attention to the individual words and their meanings differently than when engaged in a very casual conversation. People’s criteria for understanding speakers’ messages will greatly vary depending on the circumstances.

More generally, the time is ripe for scholars to incorporate task demands as an enduring part of any experimental pragmatic situation. Theories of pragmatics may need to be specifically tailored to the various tasks people perform in different experiments. It may be impossible to create comprehensive theories that supervene over experimental task demands. In this manner, the pragmatic constraints inherent in any experimental task offer another reason for claiming that pragmatics always matters in people’s experiences of language use.

## The Superficiality and Richness of Pragmatic Experience

Another challenge in conducting experimental pragmatic studies is that there are more complicated relations between task-dependent performances and pragmatic theories than are typically acknowledged. Consider a typical reading-time study that explores the cognitive effort required to understand pragmatic meaning, such as drawing a scalar implicature, inferring an ironic message, or quickly comprehending a novel metaphor. The reading time data are typically analyzed to test different hypotheses on the process by which people understand these different forms of pragmatic meaning.

However, we question whether people only infer a specific kind of meaning (e.g., literal vs. figurative, non-metaphorical vs. metaphorical, familiar metaphorical meaning vs. novel metaphorical meaning) when they read or hear language in discourse. Our motivations as readers, for example, are not simply centered on the recovery of a specific “meaning,” but involve a vast assortment of human phenomenological experiences, such as drawing more context-specific pragmatic inferences, experiencing different emotional reactions or esthetic pleasures, or imagining what you, even as an isolated participant in an experiment, may say in response to what some other person has stated. Each of these impressions, reactions, and esthetic responses may be part of the total time it takes someone to read and understand, for example, a simple metaphorical phrase as having “metaphorical” and not “literal” meaning in context.

We often fail to appreciate people’s pragmatic experiences of language in our quest to test specific hypotheses from linguistic pragmatics. To take one example, studies show that people take different times to interpret a metaphorical statement, such as “Lawyers are also sharks,” depending on whether that expression is intended to simply affirm a pre-existing belief in some discourse, add new information, or contradict a previously asserted belief (Gibbs et al., 2011). People do not simply understand a metaphor as only expressing a metaphorical meaning, but interpret it more precisely in terms of its specific pragmatic messages in context (e.g., that a speaker wishes to strengthen an existing assumption, add new information, or contradict a previously stated belief about some topic).

A different example illustrates how the amount of effort devoted to processing a speaker’s message depends on what meanings become most optimally relevant (Sperber and Wilson, 1995). For instance, reading the metaphorical phrase “My marriage is an icebox” takes longer to do in a context in which a speaker describes the state of his marriage than in a situation in which a speaker makes this reply to the question “Are you happy in your marriage? (Gibbs, 2010). The expectation set up by the prior question makes it unnecessary for readers to infer the many possible metaphorical meanings of “My marriage is an icebox” (e.g., my marriage is confining, emotionally cold, and not moving forward), precisely because the utterance quickly communicates a “no” answer to the prior “Are you happy in your marriage?” question.

Pragmatic “understanding” is not simply a matter recovering a particular type of meaning, as it also involves understanding what a speaker pragmatically, socially and esthetically intends to achieve by the use of some discourse. More attention to the exact pragmatic meanings people really infer, including their esthetic and emotional responses, in context will be an important part of broadening the vision of experimental pragmatics. We need to create experimental situations that systematically investigate when and how specific pragmatic messages are conveyed and inferred, as well as when vague, or less specific, meanings and attitudes are interpreted.

## A Case Study Example

The experimental literature on pragmatic language use is enormously complex. As noted earlier, many studies offer conflicting findings in regard to how people pragmatically produce and interpret various aspects of communicative meaning. These profound variations in experimental outcomes relate to a broader concern within psychology and elsewhere, dubbed as the “replication crisis.” Failures to replicate are now being published more than ever with some scholars claiming that any variation from some empirical standard should be interpreted as casting doubt on the validity of some earlier obtained experimental result (both for exact and conceptual replications) (Shrout and Rodgers, 2019).

We view the replication “crisis” in the behavioral sciences in a more positive light because it affords a perfect opportunity to explore all of the pragmatic nuances that shape human performances in different experimental studies. These replication problems are not problems at all, but concrete indications of how individual differences and task demands, for instance, are critical to explaining the experimental findings obtained, and why these factors are important to acknowledge in larger theories of human performance.

Consider the case of experimental research on irony understanding (Gibbs and Colston, 2007, 2012). There are many studies showing relatively fast understanding of ironic utterances in discourse, which suggests how pragmatic knowledge, of various sorts, quickly plays a role in people’s online understanding of ironic meaning (e.g., Gibbs, 1986a, b; Ivanko and Pexman, 2003). At the same time, there is data suggesting that pragmatics comes in only later on during linguistic processing when irony is encountered (e.g., Giora, 2003; Filik and Moxey, 2010). There is also considerable research on the importance of cognitive abilities related to mind-reading and executive functioning during both the learning and understanding of ironic speech and writing (e.g., Filippova and Astington, 2008).

How can we discriminate between those findings that are valid and worthy of theoretical consideration and those that are irrelevant? Replications efforts are important. Our point, though, is that replication attempts are not the solution to the diversity of experimental findings on irony comprehension, or any other pragmatic phenomena. It is far better to see the numerous experimental findings as pointing to many of the pragmatic nuances that really shape people’s complex ironic language use. For instance, many studies show that the speed with which ironic utterances are understood may vary depending on whether the experiments assessed self-paced, full statement reading time, eye-movements in which regressions back to earlier text is allowed, word-by-word moving-window measures in which regressions are not possible, paraphrase judgment times, lexical decisions to words reflecting literal or ironic meanings, judgments over whether some phrase expressed irony or not, and so on. The stimuli used in these studies included variations in the length and syntactic complexity of ironic phrases, familiar vs. novel ironic statements, different forms of irony (e.g., blame by praise vs. praise by blame), different contextual circumstances (e.g., did the context set up an ironic situation, did the context provide an explicit echo to the irony mentioned), whether the ironic statements were addressed to participants or were participants overhearers of ironic exchanges, the accent in which an ironic utterance was spoken, cases where people had to make verbal responses to ironic phrases after quickly reading them, and so on. There are also individual differences between the experimental participants in these studies which include people with different ages, language backgrounds, cultural backgrounds, occupations, personality types, organic brain disorders and injuries, different cognitive abilities (e.g., working memory capacity, mind-reading abilities), and so on. These variations in the tasks and people studied in experiments on timed irony understanding have their individual effects, but also interact in many complex ways to reveal different emergent combinations of factors that may contribute to whether verbal irony is seen as easy or more difficult to interpret.

All of these varying empirical results are subject to exact and conceptual replication attempts (and some have been replicated in one form or another). But it seems unlikely that replication efforts will somehow clean up this catalog of experimental findings to reveal a simple, comprehensive set of data which clearly points to one theoretical model of irony understanding that can be applied to all people in all situations of verbal irony use. Nonetheless, the various, sometimes complex patterns of experimental results may highlight different systems of constraint that flexibly operate to produce relevant irony interpretations in different task-specific and people-specific contexts (e.g., constraint-satisfaction models, see Campbell and Katz, 2012; Caffarra et al., 2019).

Any instance of linguistic communication fundamentally constitutes a different task for the participants given their idiosyncratic histories, dispositions, and situations. No single task captures the complex underlying psychological reality when people encounter particular combinations of word strings or utterances. Each different configuration of task demands as task constraints requires a differently self-organized mind and body. The flexible capacity to self-organize to suit task constraints exists because mind and body compose a complex system. Specifically, the embodiment of task demands constrains the mind and body to anticipate task appropriate utterances in critical states and respond as needed within an experimental setting (e.g., timed comprehension responses).

Finally, virtually all experimental studies on irony comprehension, similar to many other areas of pragmatic meaning, assume that the final product of understanding is an “ironic” message. Yet these messages vary considerably in discourse, depending on a wide range of contextual and interpersonal factors. A person may hear “A fine friend you are!” in some situation and properly infer that the speaker was not making a compliment. But the exact interpretation created is usually much more than “You are not a good friend,” and likely involves more specific meaning products, including that “the speaker had expected me to help him in my capacity as a good friend and was now scolding me with the hope that my future behaviors will be more cooperative.” All of these more nuanced pragmatic effects may be understood as part of any simple behavioral response in an experimental situation (e.g., measuring eye-movements during reading of irony in written discourse). The future challenge is to assess the relations between task-specific experimental situations and the particular, in this case, ironic messages interpreted, along with the possible emotional and affective responses of people when reading, or listening to, ironic statements. Again, the inherent complexities among people and their explicit task requirements, as well as their implicit personal motivations, may all be constitutive of pragmatics when conducting experimental pragmatic studies.

## Conclusion: Embracing a Different Theoretical Goal

These numerous challenges for experimental pragmatics may be overcome by adopting a broader vision for experimental pragmatics. There are several immediate steps toward a better understanding of the complexities of pragmatic language use.

First, researchers need to fully acknowledge the particular people they study and the implicit or explicit tasks presented to participants in experimental studies. There is no neutral point of view, no context-free, task-free environment from which utterance interpretation begins and eventually unfolds to produce pragmatic meanings. All language use is pragmatically situated from the early stages of linguistic processing, and theories of linguistic pragmatics must embrace this omnipresent reality. An experimental effect (i.e., the influence of an independent variable on a dependent variable) may be caused by a confluence of factors, most of which are not necessarily being manipulated within the context of a single study (e.g., individual differences, task demands, and the overall dynamical system that is created as a person performs in a specific task environment) (Raczaszek-Leonardi and Kelso, 2007; Gibbs and Van Orden, 2010). Experimental psycholinguistics has obtained many important empirical findings demonstrating how various pragmatic knowledge (e.g., background knowledge, contextual information, and various cognitive abilities) shape ordinary language use (Clark, 1996; Gibbs, 2019). There is still a greater need to show how the pragmatic conditions within which experimental participants operate have their influence in different facets of linguistic communication.

Second, scholars need to more fully explore the meaning products that people create when they interpret pragmatic messages in different contexts given their different understanding of goals or tasks. People do not always understand utterances in the same way, as expressing the same meanings, a fact that is true both between and within people (e.g., a single person may infer different messages from the same utterance in the same context at different times) (e.g., “good enough language comprehension,” see Ferreira and Patson, 2007). Our ultimate goal is to create a theory of pragmatics that is capable of generating the diverse meanings that people actually understand, not merely the idealized, and too often more socially and esthetically decontextualized, meanings that pragmatic theories typically discuss.

Pragmatic performances are not an isolated part of human behavior, divorced from other psychological processes and systems. People use utterances for various communicative purposes that are deeply connected with other bodily behaviors such as those responsible for tone of voice, eye-movement or gaze, laughter, bodily postures, hand and arm gestures, and so on. These bodily actions are all “coupled” in both time and space, as much cognitive science research indicates (Clark, 1996; Gibbs, 2006), to enable people to better coordinate and collaborate in order to achieve various personal and social goals (Gibbs, 2006; Shockley et al., 2009; Colston, 2019). Too much research in experimental pragmatics ignores these complex pragmatic realities when they analyze their data and go on to draw larger theoretical conclusions on the basis of the specific results they have obtained.

A general theory of pragmatics may also be characterized as part of a human dynamical system, not as its own isolated system (Gibbs, 2017). How people interpret utterances may, therefore, share many properties and processes that are related to many kinds of intentional human actions. The task that people explicitly or implicitly adopt when they produce and understand pragmatic messages, or the particular complex make-up of the participants in our studies, and the ways we analyze the full range of information that is obtained from participants are all part of the inherent pragmatic nature of human communication processes. We cannot, and should not, assume that there are ways of scrapping away the complexities in our experimental studies so that we can create a normative theory of pragmatics apart from the messy descriptive realities of real human performance.

Pragmatics is not just a temporally isolated inferential process that arises only at later points during real-life language use. Instead, pragmatics reflects the entire bodily system in action as people engage in different task-specific performances under the multiple influences of broader interpersonal, social, and cultural landscapes. Pragmatics is best understood as systems of varying constraints that have interactive influences on people’s adaptive behaviors. This broader vision embraces the view that pragmatics *always* matters, to varying degrees, and must be acknowledged, and systematically investigated, within experimental pragmatic studies.

Our call for an expanded vision of experimental pragmatics is ultimately aimed at broadening what is considered to be “pragmatics” in contemporary theories of linguistic pragmatics. Linguists and philosophers, for example, may not see questions of individual differences and task demands as being relevant to their own respective writings on pragmatic theory. However, pragmatic theories should not be divorced from the pragmatic realities of human performances. Shouldn’t these considerations of real people doing pragmatic actions be at the forefront of research and theory in linguistic pragmatics?

## Author Contributions

All authors listed have made a substantial, direct and intellectual contribution to the work, and approved it for publication.

## Conflict of Interest

The authors declare that the research was conducted in the absence of any commercial or financial relationships that could be construed as a potential conflict of interest.

## References

[B1] Abney D. Dale R. Kello C. Louwerse M. (2018). The burst and lulls of multimodal interaction: temporal distributions of behavior reveal differences between verbal and non-verbal communication. *Cogn. Sci.* 42 1297–1316. 10.1111/cogs.12612 29630740

[B2] Bara B. (2010). *Cognitive Pragmatics.* Cambridge, MA: MIT Press.

[B3] Bosco F. Tirassa M. Gabbatore I. (2018). Why pragmatics and theory of mind do not (completely) overlap. *Front. Psychol.* 9:1453. 10.3389/fpsyg.2018.01453 PMC6099790 30150960

[B4] Caffarra S. Motamed Haeri A. Michell E. Martin C. (2019). When is irony influenced by communicative constraints? ERP evidence supporting interactive models. *Eur. J. Neurosci.* 50 3566–3577. 10.1111/ejn.14503 31282038

[B5] Campbell J. Katz A. (2012). Are there necessary conditions for inducing a sense of sarcastic irony? *Discourse Process.* 49 459–480. 10.1080/0163853x.2012.687863

[B6] Clark H. (1996). *Using Language.* New York, NY: Cambridge University Press.

[B7] Colston H. (2015). *Using Figurative Language.* New York, NY: Cambridge University Press.

[B8] Colston H. L. (2019). *How Language Makes Meaning: Embodiment and Conjoined Antonymy.* New York, NY: Cambridge University Press.

[B9] Cummings L. (2019). “Clinical pragmatics,” in *Oxford Handbook of Pragmatics*, ed. Huang Y. (New York, NY: Oxford University Press), 346–361.

[B10] Ferreira F. Patson N. (2007). The ‘good enough’ approach to language comprehension. *Lang. Linguist. Compass* 1 71–83. 10.1111/j.1749-818x.2007.00007.x

[B11] Filik R. Moxey L. (2010). The on-line processing of written irony. *Cognition* 116 421–436. 10.1016/j.cognition.2010.06.005 20598677

[B12] Filippova E. Astington J. (2008). Further development in social reasoning revealed in discourse irony understanding. *Child Dev.* 79 126–138. 10.1111/j.1467-8624.2007.01115.x 18269513

[B13] Gibbs R. Jr. Colston H. (2012). *Interpreting Figurative Meaning.* New York, NY: Cambridge University Press.

[B14] Gibbs R. (1986a). Comprehension and memory for nonliteral utterances: the problem of sarcastic indirect requests. *Acta Psychol.* 53 41–57. 10.1016/0001-6918(86)90004-1

[B15] Gibbs R. (1986b). On the psycholinguistics of sarcasm. *J. Exp. Psychol. Gen.* 115 3–15. 10.1037/0096-3445.115.1.3

[B16] Gibbs R. (1994). *The Poetics of Mind: Figurative Thought, Language, and Understanding.* New York, NY: Cambridge University Press.

[B17] Gibbs R. (2006). *Embodiment and Cognitive Science.* New York, NY: Cambridge University Press.

[B18] Gibbs R. (2010). “The wonderful, chaotic, creative, heroic, challenging world of researching and applying metaphor: a celebration of the past and some peeks into the future,” in *Metaphor in the Real World*, ed. Cameron L. (Amsterdam: Benjamins), 3–17.

[B19] Gibbs R. (2017). “Metaphor and dynamical systems,” in *Routledge Handbook of Metaphor and Language*, eds Koller V. Semino E. Demjén Z. (London: Routledge), 56–70.

[B20] Gibbs R. (2019). “Experimental pragmatics,” in *Oxford Handbook of Pragmatics*, ed. Huang Y. (New York, NY: Oxford University Press), 310–325.

[B21] Gibbs R. Colston H. (eds) (2007). *Irony in Thought and Language: A Cognitive Science Reader.* Hillsdale, NJ: Erlbaum.

[B22] Gibbs R. Okonski L. Tendahl M. (2011). Inferring pragmatic messages from metaphor. *Lodz Pap. Pragm.* 7 3–28.

[B23] Gibbs R. Santa Cruz M. (2012). Temporal unfolding of conceptual metaphoric experience. *Metaphor Symb.* 27 299–311. 10.1080/10926488.2012.716299

[B24] Gibbs R. Van Orden G. (2010). Adaptive cognition without massive modularity. *Lang. Cogn.* 2 147–169.

[B25] Giora R. (2003). *On Our Mind: Salience, Context, and Figurative Language.* New York, NY: Oxford.

[B26] Hollers J. Levinson S. (2019). Multimodal language processing in human communication. *Trends Cogn. Sci.* 23 639–652. 10.1016/j.tics.2019.05.006 31235320

[B27] Huang Y. (ed.) (2019). *Oxford Handbook of Pragmatics.* Oxford: Oxford University Press.

[B28] Ivanko S. Pexman P. (2003). Context incongruity and irony processing. *Discourse Process.* 35 241–279. 10.1207/s15326950dp3503_2

[B29] Jucker A. Schneider K. Bublitz W. (eds) (2018). *Methods in Pragmatics.* Berlin: DeGruyter.

[B30] Kecskes I. (2014). *Intercultural Pragmatics.* New York, NY: Oxford University Press.

[B31] Kissine M. (2016). Pragmatics as meta-cognitive control. *Front. Psychol.* 6:2057. 10.3389/fpsyg.2015.02057 PMC4712293 26834671

[B32] McClelland J. Mirman D. Bolger D. Khaitan P. (2014). Interactive activation and mutual constraint satisfaction in perception and cognition. *Cogn. Sci.* 38 1139–1189. 10.1111/cogs.12146 25098813

[B33] McRae K. Matsuki K. (2013). “Constraint-based models of sentence processing,” in *Current Issues in the Psychology of Language: Sentence Processing*, ed. van Gompel R. (New York, NY: Psychology Press), 51–77.

[B34] Nichols S. Stich S. (2003). *Mindreading: An Integrated Account of Pretence, Self-Awareness, and Understanding Other Minds.* Oxford: Oxford University Press.

[B35] Noveck I. (2018). *Experimental Pragmatics: The Making of a Cognitive Science.* New York, NY: Cambridge University Press.

[B36] Noveck I. Sperber D. (2004). *Experimental Pragmatics.* London: Palgrave MacMillan.

[B37] Paxton A. Dale R. (2017). Interpersonal movement synchrony responds to high-and-low conversational constraints. *Front. Psychol.* 8:1135. 10.3389/fpsyg.2017.01135 28804466 PMC5532444

[B38] Raczaszek-Leonardi J. Kelso S. (2007). Reconciling symbolic and dynamic aspects of language: toward a dynamic psycholinguistics. *New Ideas Psychol.* 26 193–207. 10.1016/j.newideapsych.2007.07.003 19173014 PMC2630714

[B39] Shockley K. Richardson D. Dale R. (2009). Conversation and coordinative structures. *Top. Cogn. Sci.* 1 305–319. 10.1111/j.1756-8765.2009.01021.x 25164935

[B40] Shrout P. Rodgers J. (2019). Psychology, science, and knowledge construction: broadening perspectives from the replication crisis. *Annu. Rev. Psychol.* 69 487–510. 10.1146/annurev-psych-122216-011845 29300688

[B41] Sperber D. Wilson D. (1995). *Relevance: Communication and Cognition.* London: Blackwell.

[B42] Sperber D. Wilson D. (2002). Pragmatics, modularity and mind-reading. *Mind Lang.* 17 3–23. 10.1111/1468-0017.00186

